# On the Preparation and Medicinal Employment of Aconitine

**Published:** 1835-04-01

**Authors:** 


### ON THE PREPARATION AND MEDICINAL EMPLOYMENT OF ACONITINE, BY THE ENDERMIC METHOD, IN THE TREATMENT OF TIC DOULOUREUX AND OTHER PAINFUL AFFECTIONS. By *Alex. Turnbull*, M.D. Octavo, pp. 48.

WE are very far from wishing to discourage the trial of new and active remedies; but we begin to perceive a strong disposition to trust to *specifics*, rather than to study *indications*, and ascertain the best time and manner of applying old and established remedies. A tolerably wide range of observation and experience has taught us the superiority of the latter over the *former* species of knowledge. The practice of *specifics*, however, will always have the greatest number of followers, for very obvious reasons—but especially because it saves the necessity for labour and study. As there will ever be a considerable number of the profession who will devote themselves both to the study of indications, and to the employment of active or specific remedies, it is the duty of the journalist to direct attention to all the ways and means which human ingenuity may develop for the removal of corporeal maladies—and hence the short notice which we here insert of the puhlication under review.

It appears that there is a fear in the author's mind, lest the honour of first introducing this new poison, the aconitine, into practice, should be shared by our very talented friend and practical physician, Dr. Roots. But we encourage Dr. Turnbull not to despair. If aconitine prove doubly more useful than veratria, the Doctor will not lose posthumous fame by the omission of his name in conjunction with either remedy. We think we may safely say, that veratria is gone to the tomb of the Capulets—and that it will only be remembered, ten years hence, by those who have smarted under its effect without benefit, or suffered in their pockets by the expense of its purchase. Its promulgator will not accuse us of throwing cold water on the discovery, nor ought he to complain if, with even-handed justice, we declare our conviction, that the veratria will soon cease to be a remedy in the hands of the judicious practitioner. In the same course of honest, well-meant, and independent principles, we proceed to make our brethren acquainted with the new candidate for remedial fame.

### Preparation.

" A quantity of the fresh root of the Aconitum Napellus must be procured, and care should be taken that it be sound, and that the root be that of monkshood; for sometimes other roots are sold for it. Let it be carefully and cautiously dried, and then reduced to powder; this latter operation is not unattended by danger, especially if a part of the fine dust which rises from it be inhaled. One part by weight of the powder, and two parts by measure of strong alcohol, are to be digested together in a gentle heat for seven days, and the tincture, while warm, is to be filtered. It is then to be reduced to the consistence of an extract, by careful evaporation, at a low and well-regulated temperature; the object of this, is to prevent the destruction or expulsion of the active principle, which would very probably ensue, if the temperature employed were higher than barely sufficient to carry off the alcohol. To the extract thus prepared, liquid ammonia is to be added, drop by drop, and mixed well with it, to precipitate the alcaloid; and in this part of the process, care must be taken that too much be not added, as in some instances the product appears to have been decomposed by inattention to this circumstance. It is difficult to give a precise rule as to the quantity; but enough will have been added, if the extract give out the odour of ammonia, when stirred.

The mass now consists of impure Aconitine, mixed up with a quantity of extractive and other matters, soluble in water; and it may be taken up either with boiling alcohol, or sulphuric ether; or the soluble matter may be removed, by repeated washings with small quantities of cold water, which will leave the Aconitine. This latter process is the one we have generally employed, and is performed by pouring a little water on the extract, and mixing them carefully together, then allowing the undissolved part to subside, pouring off the fluid, and repeating the operation, as long as any soluble matter is taken up, a quantity of light brown or gray powder is left, which may be purified by subsequent solution in alcohol. This powder contains the active properties of the Aconite, in a high degree of concentration. A grain of it was dissolved in a drachm of alcohol; and twenty drops of the solution put into the mouth of a guinea-pig, occasioned death in a few minutes. Other experiments have been performed; all of which prove the extreme energy of the substance.

The second process consists in dissolving the alcoholic extract, prepared as before, without the addition of the ammonia, in as much cold water as will take it up, and carefully decanting the solution from the insoluble part, and then filtering it. To the filtered solution, liquid ammonia is to be added, drop by drop, as long as it occasions any precipitation. When the precipitate has subsided, the supernatant fluid should be carefully poured away, or drawn off by means of a syphon; and after the precipitate has been deprived of as much of the fluid as possible, it should be purified by a sufficient number of washings with small quantities of cold water, and then carefully dried. The product obtained by this process is white." 15.

We would advise those who mean to test the aconitine, to procure it from Mr. Morson, as the process of preparation is far from being an easy one. The action of the new medicine on the skin is said to be weaker than that of either veratria or delphinia—" for in no case, hitherto observed, has it produced a greater degree of vascular excitement than might easily be accounted for *by the friction itself*." It is by no means impossible that this remark might be extended to more than the *vascular system*. The diseases to which Dr. T. has applied the aconitine, are tic douloureux and neuralgic affections generally. But, after the magic cures performed by the veratria, why employ the aconitine! The formula is as follows :—

℞. Acontinæ, gr. ij.

Alcohol, gtt. vj. tere bene.

Et adde Axungiæ, ℥j. Misce ft Unguentum.

The proportion of the active ingredient may be gradually increased.

One or two imperfect cases are related by Dr. Turnbull himself, and one reprinted from the Medical and Surgical Journal, for December 13th, 1834, which was treated by Dr. Roots at St. Thomas's Hospital. To these we may refer those who are desirous of giving a " fair trial" to the new candidate for fame and favour.

